# Cell Cycle Regulated Interaction of a Yeast Hippo Kinase and Its Activator MO25/Hym1

**DOI:** 10.1371/journal.pone.0078334

**Published:** 2013-10-21

**Authors:** Jonathan Hsu, Eric L. Weiss

**Affiliations:** Department of Molecular Biosciences, Northwestern University, Evanston, Illinois, United States of America; Texas A&M University, United States of America

## Abstract

Hippo pathways are ancient signaling systems that contribute to cell growth and proliferation in a wide diversity of eukaryotes, and have emerged as a conserved regulator of organ size control in metazoans. In budding yeast, a Hippo signaling pathway called the Regulation of Ace2 and Morphogenesis (RAM) network promotes polarized cell growth and the final event in the separation of mother and daughter cells. A crucial regulatory input for RAM network control of cell separation is phosphorylation of a conserved hydrophobic motif (HM) site on the NDR/LATS family kinase Cbk1. Here we provide the first direct evidence that the Hippo-like kinase Kic1 in fact phosphorylates the HM site of Cbk1, and show that Kic1 is allosterically activated by Hym1, a highly conserved protein related to mammalian MO25. Using the structure of mammalian MO25 in complex with the Kic1-related pseudokinase STRAD, we identified conserved residues on Kic1 that are required for interaction with Hym1. We find that Kic1 and Hym1 protein levels remain constant throughout the cell cycle but the proteins’ association is regulated, with maximal interaction coinciding with peak Cbk1 HM site phosphorylation. We show that this association is necessary but not sufficient for this phosphorylation, suggesting another level of regulation is required to promote the complex to act upon its substrates. This work presents a previously undiscovered cell cycle regulated interaction between a Hippo kinase and a broadly conserved allosteric activator. Because of the conserved nature of this pathway in higher eukaryotes, this work may also provide insight into the modularity of Hippo signaling pathways.

## Introduction

 Hippo pathways in yeast comprise core elements of an ancient signaling pathway that contributes to cell proliferation and morphogenesis in various eukaryotes. First identified in *Drosophila melanogaster*, the Hippo/MST signaling pathway includes a set of genes responsible for controlling tissue size by regulating cell proliferation and cell death [1-5]. Mutations in these genes have been shown to cause abnormal cell growth and decreased cell death, suggesting that disruptions in the Hippo pathway can lead to tumorigenesis. Recently, numerous studies have shown similar phenotypes in humans and mice, classifying the Hippo pathway as a tumor suppressor signaling pathway [1,6,7].

 In Hippo pathways, germinal center kinases (GCKs) like MST/Hippo are thought to directly control AGC kinases like NDR/LATS (nuclear Dbf2-related/large tumor suppressor), which form a critical association with the highly conserved MOB family coactivator. The NDR/LATS kinase-MOB coactivator complex regulates cell proliferation and morphogenesis through phosphorylation of transcription factors. In budding yeast, this pathway is related to the RAM network (Regulation of Ace2 and Morphogenesis) and the mitotic exit network (MEN), both of which contain components that include a Hippo-like kinase and an NDR/LATS kinase [8].

 The budding yeast NDR kinase, Cbk1, contains crucial regulatory phosphorylation sites in its activation loop (T-loop) and C-terminal hydrophobic motif (HM). Both of these regulatory sites must be phosphorylated for full activation of the NDR/LATS kinase, as mutation of either phosphoacceptor residues to alanine generally abolishes kinase activity [9-13]. Previous studies in mammals have shown that MST kinases specifically phosphorylate the HM site of an NDR kinase [9,12], and the MEN Hippo-like kinase Cdc15 in budding yeast can directly phosphorylate the NDR-like kinase Dbf2 [10]. Also, we have previously observed Cbk1 HM site phosphorylation that is abolished upon deletion of RAM network components, including the Hippo-like kinase Kic1 [14]. Phosphorylation of this HM site is regulated through the cell cycle, peaking at times of cell separation; intriguingly, only a small fraction of the kinase (approximately 3-5%) is phosphorylated at this site at peak times [15]. Misregulation of the HM site leads to failure of cells to separate, suggesting that proper cell cycle timing of phosphorylation and activation of Cbk1 is critical for proper cell separation.

 How then is the Cbk1 HM site regulated? Previous structural studies of mammalian Hippo MST kinase associated with the scaffold protein MO25 have shown that MO25 acts as an allosteric activator of the kinase [16]. MO25 is a highly conserved helical armadillo-repeat protein and is related to the yeast RAM network protein Hym1. Previous studies of orthologs of MO25 have shown that it can interact with and activate MST-like kinases, suggesting that MO25 may have a role in Hippo signaling [17-20].

 In this study, we examine the MO25 ortholog in *S. cerevisiae* Hym1 and characterize its interaction with the Hippo-like kinase Kic1. We demonstrate that Hym1 can bind to Kic1 and allosterically activate the kinase to specifically phosphorylate the HM site of the NDR kinase Cbk1. The association of this complex depends on conserved interaction motifs that closely resemble that of the MST-related pseudokinase STRAD and MO25. We also show a novel regulated interaction between Hym1 and Kic1, where peak association occurs when we observe peak HM site phosphorylation. Our findings suggest that the complex of MO25 proteins with MST/Hippo kinases is an ancient regulatory module, the formation of which was critical for activating Hippo signaling in early eukaryotes.

## Materials and Methods

### Recombinant protein expression and purification

We cloned a catalytically inactive Cbk1 (residues 251-756, D475A) and full length Mob2 into a bicistronic expression system. We coexpressed the N-terminally hexahistidine (His_6_)-tagged Cbk1 and N-terminally GST-tagged Mob2 in *E. coli* Rosetta(DE3)pLysS. We copurified the proteins by chromatography on Ni-nitrolotriacetic acid resin (Qiagen) followed by glutathione-Sepharose 4B (GE Healthcare) using the manufacturer’s protocols. The proteins were dialyzed into 50 mM Tris, 100 mM NaCl, 1 mM dithiothreitol (DTT), pH 8.0, concentrated, flash-frozen in liquid nitrogen, and stored at -80°C.

We cloned full length Hym1, a fragment of Kic1 (residues 1-359), and a C-terminal Cbk1 fragment (residues 673-756) into a modified pET vector containing N-terminal GST and C-terminal His_6_ tags. We also constructed point mutation alleles for both the Kic1 (D57A, L90Q, C134F, and W357A/D358A/F359A) and Cbk1 (T743E) recombinant proteins. All of these fusion proteins were expressed in *E. coli* Rosetta(DE3)pLysS and purified in the same manner as the His_6_-Cbk1(251-756, D475A):GST-Mob2 complex. For recombinant Hym1 purification, however, we dialyzed the protein into 10 mM Tris, 100 mM NaCl, 2 mM DTT, pH 7.4 and added Tobacco Etch Virus (TEV) protease to cleave the GST tag. The protein was then dialyzed again into 50 mM Tris, 100 mM NaCl, 1 mM DTT, pH 8.0, concentrated, flash-frozen in liquid nitrogen, and stored at -80°C.

For all purified recombinant proteins, we measured the optical density at 280 nm by using a NanoDrop spectrophotometer, and determined the concentration using the protein’s extinction coefficient, calculated based on its amino acid composition.

### *In vitro* kinase assays

 For Kic1-Hym1 kinase assays, we used equimolar (1 µM) GST-Kic1(1-359)-His_6_ and Hym1-His_6_. For Hym1 titration assays, we used 0.5 µM GST-Kic1(1-359)-His_6_ and indicated amounts of Hym1-His_6_. In all kinase assays involving Kic1 and Hym1, we pre-incubated GST-Kic1(1-359)-His_6_ with Hym1-His_6_ in 50 mMM Tris (pH 8.0) and 100 mM NaCl for 1 h at room temperature. We then combined Kic1-Hym1 with 10 µM GST-Cbk1(673-756)-His_6_ fragment as the substrate in a 15-µl reaction mixture containing 50 mM Tris (pH 8.0), 100 mM NaCl, 10 mM MgCl_2_, 1 mM MnCl_2_, 1 mM DTT, 1 µM ATP, and 5 µCi [γ-^32^P]ATP. We incubated reaction mixtures at room temperature for the indicated amount of time and quenched the reactions by addition of 5× SDS-PAGE sample buffer with immediate mixing followed by 10 min of incubation at 85°C. We resolved proteins on a 12% SDS-PAGE gel, and then stained with Coomassie Blue. We evaluated the extent of Cbk1 labeling by using a Storm 860 Phosphorimager (Molecular Dynamics) to quantify the amount of ^32^P incorporated into Cbk1. We measured pixel intensities by using ImageQuant software (Molecular Dynamics) and compared volume over gel background in regions of interests encompassing the Cbk1 bands with regions of interests encompassing simultaneously acquired spots of known ^32^P quantities.

### Yeast strains, plasmids, and methods

 All strains generated and used in this study are derived from the BY4741 genetic background. We generated deletion, hemagglutinin (HA)-tagged, and myc-tagged strains by standard gene replacement or tagging [21]. We cultured cells in YPD (1% yeast extract, 2% peptone, and 2% glucose). 

 We constructed centromeric Kic1 and Hym1 plasmids by cloning full length *KIC1* and *HYM1* coding sequence along with sequences 500 bp upstream and downstream of the start and stop codons from genomic DNA into pRS315 and pRS316 vectors. To generate *kic1* alleles, we introduced substitutions by site-directed mutagenesis using *Pfu* Turbo (Agilent). We constructed the Hym1-Kic1 fusion construct by cloning the Hym1-13myc sequence 5’ to the Kic1-3HA in a pGREG506 vector [22] with an *ADH1* promoter, resulting in pGREG506-P_*ADH1*_-*HYM1*-13myc-*KIC1*-3HA. We introduced plasmids by standard lithium acetate transformation and cultured cells in synthetic minimal selection medium (0.67% yeast nitrogen base without amino acids, 2% glucose, and 0.2% amino acid drop in; US Biological).

 For QPCRs, we prepared RNA from log-phase cultures by hot acid phenol extraction and performed quantitative RT-PCRs using *CTS1* and *ACT1* primers as previously described [15].

### Cell synchronization

 For the *GAL-CDC20* synchrony experiments, we grew cells to log phase at 30°C in either YP or synthetic minimal media + 2% galactose, filtered, and resuspended into an equal volume of YP + 2% glucose and continued to grow the cells at 30°C for 2.5 h until M phase arrest (assessed by accumulation of large-budded cells). We filtered the cells again, resuspended them into YP + 2% galactose, grew at 30°C, and removed samples at indicated time points. We harvested cells by centrifugation, fixing a small fraction in 3.7% formaldehyde for microscopy of budding index and then freezing the remainder in liquid nitrogen.

### Co-immunoprecipitation

For Kic1-Hym1 co-immunoprecipitations, we prepared cell lysates by bead beating as previously described [14], and immunoprecipitated with anti-c-Myc mAb (9E10, a gift of M. Glotzer, University of Chicago) using Dynabeads Protein G (Life Technologies) following the manufacturer’s standard protocols. For analysis of Cbk1 HM site phosphorylation, we immunoprecipitated Cbk1 with anti-Cbk1 and protein G-Sepharose (Life Technologies) as previously described [15], using a fraction of the cell lysate from the Kic1-Hym1 co-immunoprecipitations. For all immunoprecipitations, we resuspended the beads in 2× SDS-PAGE sample buffer and resolved the samples on 10% SDS-PAGE gels.

### Immunoblotting

 For all Western blot assays, we transferred proteins to Immobilon-FL polyvinylidene difluoride (PVDF; Millipore) membrane. We blocked the membranes for 30 min at room temperature in Odyssey blocking buffer (LI-COR). We probed for Kic1-HA with 1:1000 anti-hemagglutinin (HA) mAb (12CA5, a gift of R. Lamb, Northwestern University) and Hym1-myc with 1:1000 rabbit polyclonal anti-c-Myc (Rockland). We probed for phosphorylated Cbk1 HM site with 1:500 affinity-purified phosphospecific rabbit antibody (anti-pT743) [14]. We incubated primary antibodies with blots for 1 h at room temperature, washed, and then incubated with 1:20,000 goat anti-mouse IRDye 680LT secondary (LI-COR) or 1:15,000 goat anti-rabbit IRDye 800CW secondary (LI-COR) for 30 min. We washed the blots and imaged with an Odyssey fluorescent imaging system (LI-COR) and quantified with ImageQuant software (Molecular Dynamics).

### Microscopy and image analysis

 We grew cells to mid-log phase performed differential interference contrast (DIC) microscopy using an Axiovert 200 M microscope (Carl Zeiss MicroImaging, Inc.) with a Cascade II-512B camera (PhotoMetrics, Inc.). We captured images with a 100×/1.45-numerical aperture oil immersion objective using the Openlab software (Improvision).

## Results

### Kic1 directly phosphorylates Cbk1 at its conserved hydrophobic motif site

**Figure 1:**
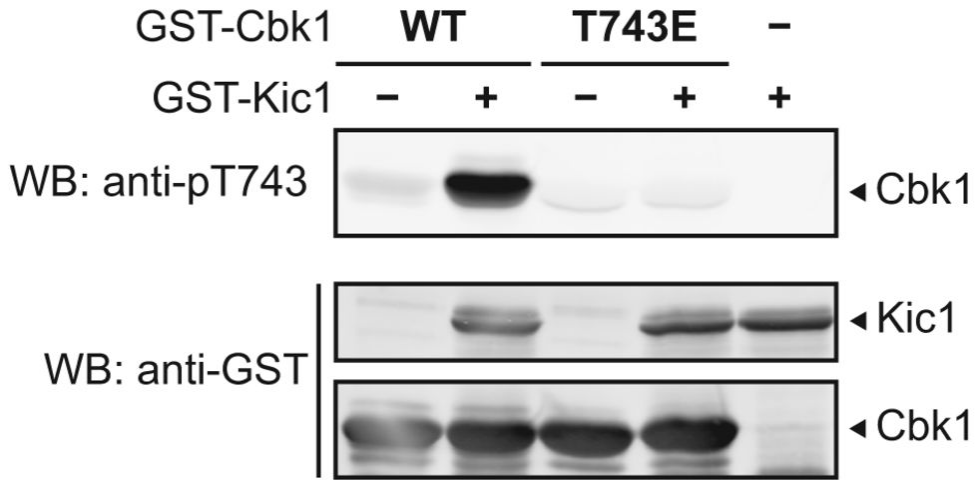
Kic1 directly phosphorylates the Cbk1 HM site *in* *vitro*. We performed kinase assays with bacterially purified recombinant GST-Kic1(1-359)-His_6_ as the kinase and GST-Cbk1(673-756)-His_6_ as the substrate where the phosphoacceptor site Thr743 was either WT or mutated to a non-phosphorylatable residue (T743E). When we used a phosphospecific antibody that recognizes HM site phosphorylation, we saw specific phosphorylation of the HM site only when the residue is WT and not mutated (top). We used equal amounts of Kic1 as well as Cbk1 between reactions as indicated, which we assessed with anti-GST (bottom).

Like other NDR/LATS kinases, the budding yeast protein kinase Cbk1 is controlled by a highly conserved HM site located at the C-terminal region of the kinase [11,14,23,24]. In metazoans, MST/Hippo family kinases have been shown to phosphorylate NDR/LATS kinases [12,25]. Furthermore, in budding yeast, the Hippo-related kinase Cdc15 has been well established to phosphorylate Dbf2, a closely related substrate to Cbk1 [10]. Genetic evidence strongly suggests that Kic1 functions upstream of Cbk1 by directly phosphorylating Cbk1’s HM site [26,27]; we sought direct evidence to confirm this. Using a previously developed phosphospecific antibody that recognizes the phosphorylated threonine of the HM site [14,15] we found that a bacterially purified fragment of Kic1 containing the kinase domain phosphorylated the wild-type HM site of a GST-tagged Cbk1 peptide containing the C-terminal region (amino acids 673-756) *in vitro* (Figure 1). This result does not rule out other potential phosphoregulation sites on the C-terminal region of Cbk1, as we observe additional phosphorylation via autoradiograph even when the HM site is mutated to a non-phosphorylatable residue (Figure S1). 

### Hym1 enhances Kic1 kinase activity and Cbk1 HM site phosphorylation

**Figure 2:**
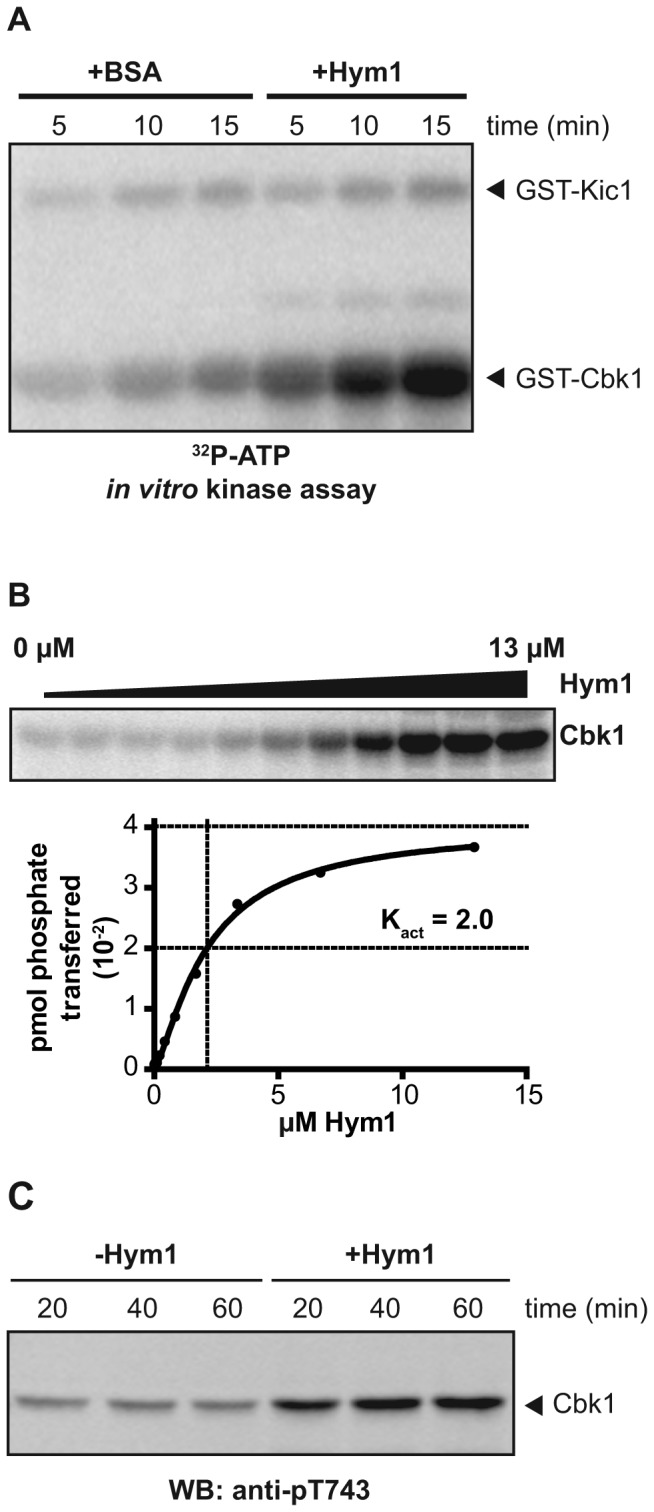
Hym1 enhances Kic1 kinase activity *in* *vitro*. **A**. We performed kinase assays with bacterially purified recombinant GST-Kic1(1-359)-His_6_ as the kinase and GST-Cbk1(673-756)-His_6_ as the substrate, using γ^32^P-ATP. Where indicated we added bacterially purified recombinant full-length Hym1-His_6_, or BSA as a control. Cbk1 phosphorylation is significantly increased when Hym1 was present, compared to when Hym1 was absent. **B**. To determine if Hym1 binding to Kic1 is an allosteric effect, we titrated the amount of Hym1 while keeping Kic1 concentrations constant. Starting at 13 µM, we made 2-fold serial dilutions of Hym1 and performed kinase assays and measured amount of phosphate transferred to the GST-Cbk1(673-756)-His_6_ substrate. Kic1 kinase activity (phosphate transferred) was dependent on Hym1 concentration; we determined that half maximal activation (K_act_) of Kic1 occurred at approximately 2.0 µM Hym1. **C**. We performed kinase assays with bacterially purified recombinant GST-Kic1(1-359)-His_6_ as the kinase and His_6_-Cbk1(272-756,D475A):GST-Mob2 as the substrate. Where indicated, we added bacterially purified recombinant full-length Hym1-His_6_. When Hym1 was present, we observed a significant increase in phosphorylated Cbk1 HM site as detected by anti-pT743.

Kic1 was identified as an interacting partner of the helical repeat protein Hym1, which is closely related to mammalian MO25 [27-30]. Structural analysis of MO25 in complex with the catalytically inactive Kic1-related pseudokinase STRAD, as well as several catalytically active mammalian MST/Hippo-related kinases, suggested that MO25 proteins function as allosteric activators of bound kinases [18,31,32]. In yeast, deletion of *HYM1* resulted in a loss of Cbk1 HM site phosphorylation as well as a cell separation defect, phenocopying *cbk1∆* [14,27,28]. To further understand the function of Hym1’s association with Kic1 and the role of this complex in HM site phosphorylation we purified recombinant full-length Hym1 and introduced it into kinase assays with recombinant Kic1 and the Cbk1 C-terminal peptide (amino acids 673-756) fused to GST. We found that addition of Hym1 dramatically increased phosphorylation of the GST-Cbk1 tail substrate (Figure 2A). Furthermore, we found that increasing the concentration of Hym1 resulted in increased Kic1 kinase activity (Figure 2B). We determined that half maximal activation of Kic1 occurred at approximately 2.0 µM Hym1 (termed K_act_), which is comparable to the equilibrium binding constants of mammalian MO25 and MST kinases (2-10 µM) [18].

 Because Cbk1 is only functional when bound to its co-activator Mob2 [33], we assessed Kic1-Hym1 phosphorylation of Cbk1’s HM site when Cbk1 is complexed to Mob2. We found that the HM site of recombinant catalytically inactive Cbk1 containing amino acids 251-765 associated with full length Mob2 was more phosphorylated when Hym1 is present compared to when Hym1 is absent (Figure 2C). These *in vitro* results suggest that, like the mammalian MO25-STRAD complex, Hym1 acts as an allosteric activator to Kic1. While Kic1-Hym1 clearly phosphorylated the Cbk1 HM site, this was only robustly detectable using the anti-pT743 antibody, and overall our findings suggest that the HM site is relatively inefficiently phosphorylated in the context of the Cbk1-Mob2 complex compared to a short peptide of Cbk1.

### The mechanism of Hym1 interaction with Kic1 is conserved

**Figure 3:**
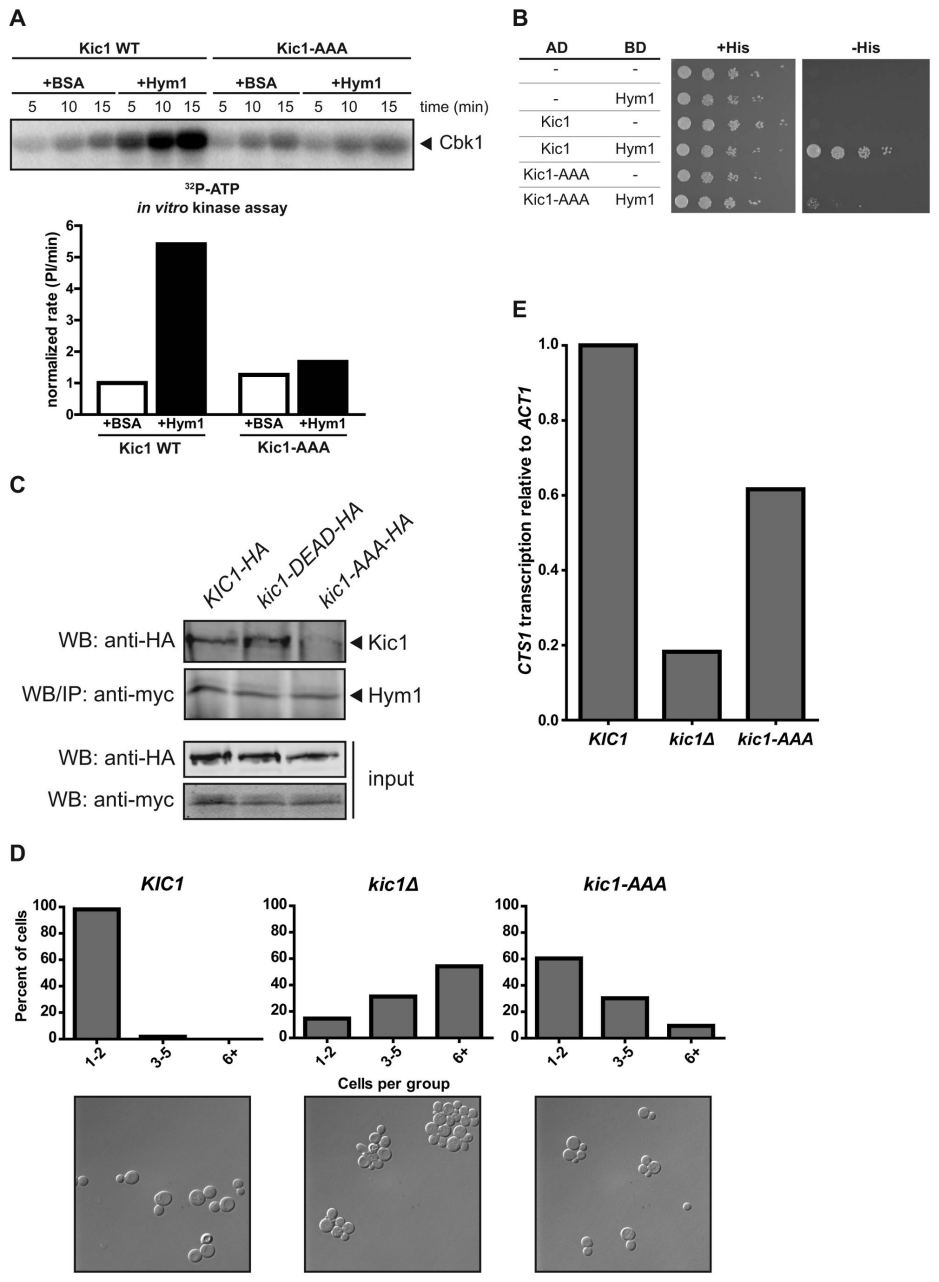
The WDF motif on Kic1 is important for activation by Hym1. **A**. We performed kinase assays with bacterially purified recombinant GST-Kic1(1-359)-His_6_ where the Trp-Asp-Phe (WDF; residues 357-359) motif on Kic1 was either WT or mutated to AAA as the kinase and GST-Cbk1(673-756)-His_6_ as the substrate using γ^32^P-ATP. Where indicated, we added bacterially purified recombinant full-length Hym1-His_6_ or BSA as a control. We measured the reaction rates over a 15 min kinase reaction and normalized the rates to Kic1 WT in the absence of Hym1. When Hym1 was present, we observed a 5-fold increase in reaction rate. When the WDF motif was mutated to AAA, however, we observed a loss of the ability for Hym1 to activate Kic1 kinase activity. **B**. We performed a yeast two-hybrid analysis to confirm that the WDF motif on Kic1 is important for Kic1-Hym1 interaction. We cloned Kic1 alleles and Hym1 into pGAD-C1 and pGBD-C1, and we coexpressed these constructs as fusion proteins with the GAL4 activation domain and DNA-binding domain, respectively. We used plasmids expressing the indicated proteins either as prey or bait alone as negative controls and wild type Kic1 and Hym1 as a positive control. We found that the WDF mutant abolished Kic1-Hym1 interaction. **C**. When we performed co-immunoprecipitation of myc-tagged Hym1 from yeast cell extracts, we were able to recover HA-tagged wild type Kic1 as well as a catalytically inactive Kic1 allele (kic1-DEAD), but not the Kic1 WDF mutant (kic1-AAA), suggesting the importance of the WDF motif for the Kic1-Hym1 interaction. **D**. We quantified cell separation defects in *kic1* mutant strains, shown as the percentage of groups of cells in the population in clumps of the indicated number of connected cells. **E**. We measured the expression of the septum degrading enzyme *CTS1* by quantitative RT-PCR, and we found that *kic1-AAA* was defective for transcriptional activity but not the extent of *kic1Δ*.

Given that Hym1 is highly similar to the mammalian MO25 protein and can enhance Kic1 kinase activity, we hypothesized that the two pathways likely have similar biochemical mechanisms. Previous studies have shown that MO25 binds to a Kic1-related pseudokinase STRAD as well as several MST/Hippo kinases [18,34]. MO25 association causes STRAD to adopt an “active-like” kinase conformation [32]. This complex then engages the tumor suppressor LKB1 to form an active ternary complex [31]. Crystal structure of MO25 in complex with STRAD was used to identify key residues important for the interaction, specifically, a conserved tryptophan and phenylalanine residue, termed site D [18,32]. We hypothesized that the Kic1-Hym1 interaction would resemble that of the MO25-STRAD complex. To test this hypothesis, we produced and purified a recombinant GST-tagged Kic1 kinase domain construct (amino acids 1-359), containing mutations in the site D (WDF to AAA) motif. We assayed the ability of this Kic1 allele to phosphorylate the GST-Cbk1 tail substrate (amino acids 673-756) in the presence or absence of recombinant full length Hym1. There was over 5-fold increase in wild-type Kic1 kinase activity in the presence of Hym1 relative to the activity in the absence of Hym1. The activity of the site D allele of Kic1, however, showed no apparent increase upon addition of Hym1 (Figure 3A). Interestingly, site D is a highly conserved motif C-terminal to the kinase catalytic domain that is present in almost all orthologs of MST/Hippo kinases [18,19]. We also tested three other interaction sites (sites A-C) identified in MO25-STRAD, and found that only site A appeared to reduce kinase activity when mutated (Figure S2).

 We further assessed the conserved WDF interaction motif in Kic1 using two-hybrid analysis. As previously reported, we observed two-hybrid interaction between wild type Kic1 and Hym1, but found that the interaction was greatly reduced in the site D mutant (Figure 3B). The site A mutant allele also abolished two-hybrid interaction but we were unable to assess protein levels of this allele (Figure S2). We were also able to abrogate Kic1-Hym1 co-immunoprecipitation *in vivo* when we made this mutation endogenously (Figure 3C). Interestingly, a catalytically inactive allele of Kic1 was still able to co-immunoprecipitate with Hym1, indicating that the interaction is independent of Kic1 kinase activity. 

Next, we wanted to understand the function of this interaction site *in vivo*. It has been shown that cells lacking Kic1 are unable to phosphorylate the Cbk1 HM site and have cell separation defects because Cbk1 cannot turn on transcription of septum-degrading enzymes like *CTS1* [14,27]. We observed this separation defect in *kic1∆* cells and were able to rescue it in the presence of a wild-type *KIC1 CEN* plasmid. Cells containing the site D mutant allele, however, exhibited only partially defective cell separation presumably due to decreased *CTS1* transcript levels (Figure 3D-E). Taken together, our mutational analysis of Kic1 indicates that it interacts with Hym1 in a mechanism similar to MST/Hippo and MO25. Notably, our work indicates that site D is a critical feature of Hippo-related kinases that promote interaction with MO25/Hym1 to allow proper activation of the kinase. By analogy with the MO25-STRAD interaction, it is likely that Hym1 association brings Kic1 into an active conformation, where the residues on the αC-helix interact with the concave surface of Hym1 [32].

### Kic1 and Hym1 interact in a regulated manner

**Figure 4:**
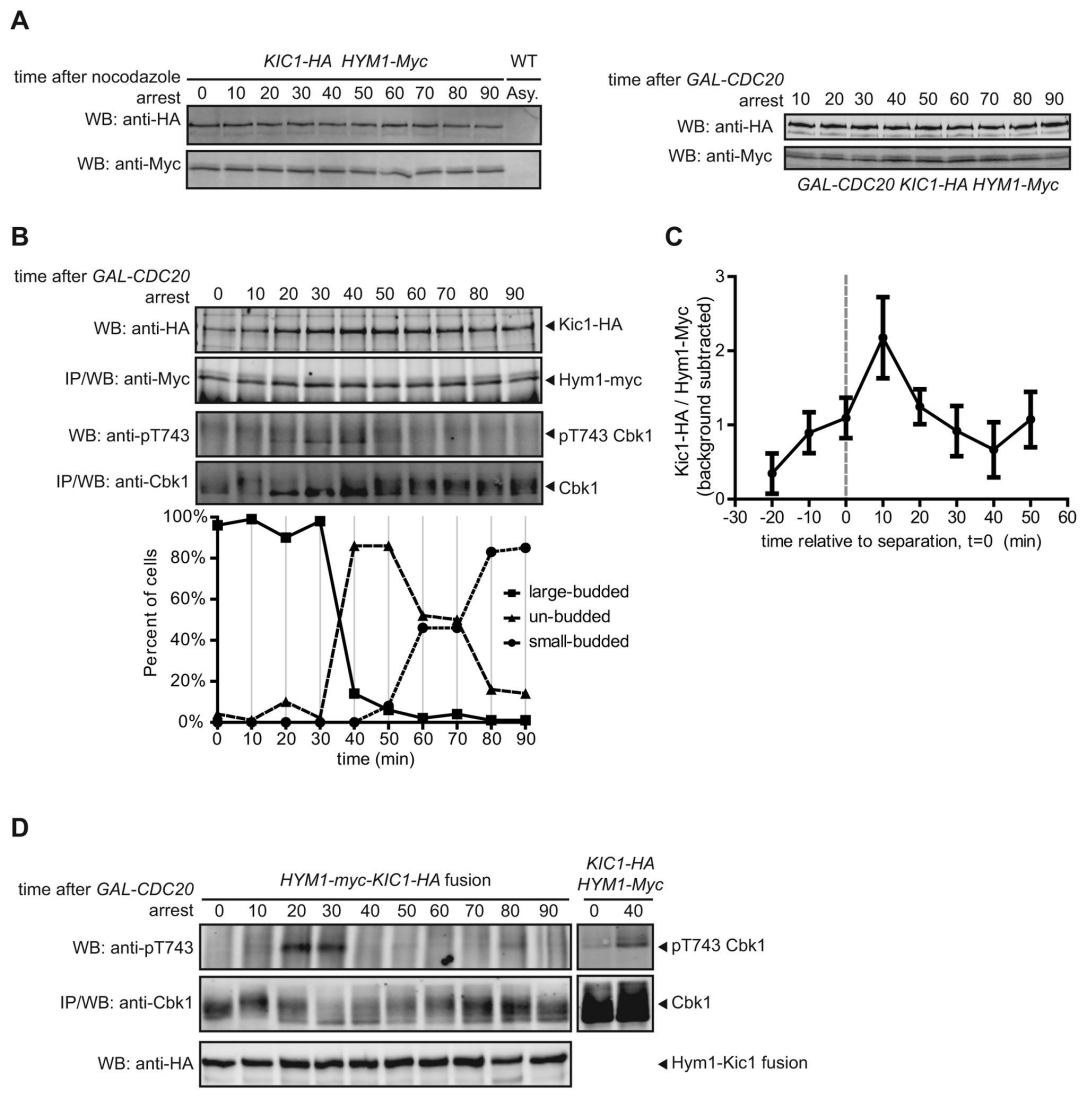
Kic1 and Hym1 interact in a regulated manner. **A**. We prepared total cell lysates from cells arrested and released from nocodazole as well as *GAL-CDC20*. Both HA-tagged Kic1 and myc-tagged Hym1 protein levels remained constant throughout the release. **B**. We performed co-immunoprecipitations of myc-tagged Hym1 at 10 min intervals upon release from *GAL-CDC20* arrest and recovered HA-tagged Kic1. The levels of Kic1-HA that we co-immunoprecipitated varied, starting low and peaking around 40 min after release. We also probed immunoprecipitated Cbk1 with an anti-pT743 antibody and observed peak HM site phosphorylation coinciding with peak Kic1-HA co-immunoprecipitation. We quantified budding index (large-, small-, or un-budded) at each time point. Blots and budding index represent one of four trials. **C**. To quantify co-immunoprecipitation data, we measured pixel intensities of both Kic1-HA and Hym1-myc at each time point and took the ratio of Kic1-HA to Hym1-myc. Within each trial, we treated the time point with the lowest ratio as background (generally t=0) and subtracted this from all other time points. We adjusted each trial by setting t=0 to when we see a precipitous drop in large-budded cells, which correlates with the start of cell separation. We then averaged each of the time points and show standard error of the mean of four independent trials. We consistently see a peak of Kic1-Hym1 co-immunoprecipitation 10 min after separation. **D**. We probed immunoprecipitated Cbk1 with an anti-pT743 antibody at 10 min intervals upon release from *GAL-CDC20* arrest in cells expressing a Hym1-Kic1 fusion. We observe that HM site phosphorylation remains regulated through the cell cycle even when cells express a Hym-Kic1 fusion. As a control, we probed for HM site phosphorylation at t=0 (no HM site phosphorylation) and t=40 (maximal HM site phosphorylation) in WT cells. We also observed Hym1-myc-Kic1-HA fusion protein levels remained constant throughout the release.

Cbk1 HM site phosphorylation is a regulated event, occurring maximally following mitotic exit as mother and daughter cells are separating [15]. Since the Kic1-Hym1 complex directly phosphorylates the Cbk1 HM site (Figure 1), we hypothesized that regulation of the interaction between this MST/Hippo-related kinase and its activator helps achieve this temporal control. One manner in which this could occur is periodic synthesis and degradation of either protein, analogous to the production and regulated proteolysis of cyclin in the control of CDK function [35]. We found, however, that both Hym1 and Kic1 protein levels remained constant throughout the cell cycle upon release from two different metaphase/anaphase arrests (Figure 4A). Therefore, the activity of the Kic1-Hym1 complex is not controlled by regulated synthesis or destruction of either protein.

To determine if the Kic1-Hym1 interaction is cell cycle regulated we synchronized a strain containing Kic1-3HA and Hym1-13myc using arrest and release from a metaphase/anaphase block [36] and assessed co-immunoprecipitation of Kic1 and Hym1 at 10 minute intervals. Since synchrony varied slightly from experiment to experiment, we quantified the bud size (large, none, or small) at each time point to establish a budding index of each trial [37-39]. We used the start of cell separation (when the percentage of large-budded cells dropped) as a reference point between trials (Figure 4B). We found that Kic1-Hym1 co-immunoprecipitation levels consistently peaked as cells began to separate (Figure 4C). This result suggested that Kic1 could be most active at this time and thus Kic1 substrates, specifically Cbk1, would be maximally phosphorylated. Thus, we examined HM site phosphorylation of Cbk1’s HM site through the release. We saw peak HM site phosphorylation 10 minutes after cells began separating, coinciding strongly with peak Kic1-Hym1 association (Figure 4B). 

**Figure 5:**
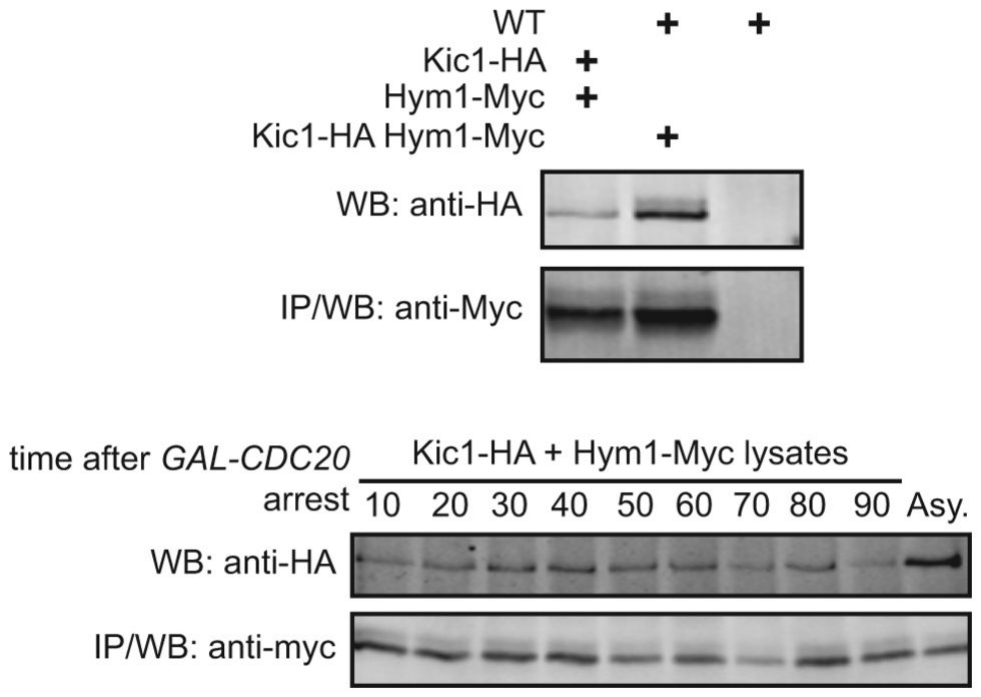
Kic1 and Hym1 co-immunoprecipitate from separate lysates. We prepared cell lysates of WT, Kic1-HA, Hym1-Myc, and Kic1-HA Hym1-Myc separately. We mixed lysates as indicated and performed co-immunoprecipitation of Hym1-Myc and recovered Kic1-HA. We found that there was more Kic1-HA associated with Hym1-Myc when we used lysate from a strain that contained both Kic1-HA and Hym1-Myc. We also prepared separate lysates of Kic1-HA and Hym1-myc at 10 min intervals upon release from *GAL-CDC20* arrest. We mixed these lysates and performed co-immunoprecipitation of Hym1-Myc and recovered Kic1-HA. We found a significant association of Kic1 and Hym1 that is likely contributing to a basal level of interaction between these two proteins in our co-immunoprecipitation experiments.

Co-immunoprecipitation experiments that seek to quantify protein complex formation *in vivo* are subject to two types of artifacts: (I) interaction of proteins following lysis to form complexes not present in the cells and (II) dissociation of complexes present in live cells during isolation. Since the Kic1-Hym1 interaction is likely in the low micromolar range, there is probably significant dissociation during immunoprecipitation that is difficult to quantify. Although co-immunoprecipitation of Kic1-HA with Hym1-myc clearly peaked during cell separation in multiple experiments, we saw a consistently high level of basal Kic1-Hym1 interaction throughout the time courses. To determine if this could be attributed to post-lysis association we prepared lysates from cells expressing either Kic1-3HA or Hym1-13myc separately, mixed them, and then assessed Kic1-HA co-immunoprecipitation with Hym1-myc. We saw a significant level of co-immunoprecipitation, demonstrating that post-lysis association probably contributes to the basal level of interaction between these two proteins in our experiments. Importantly, however, we did not see an increase in post-lysis Kic1-Hym1 association in synchronized cells, further supporting the regulated formation of this complex in vivo following mitotic exit (Figure 5).

### HM site phosphorylation requires additional control

Since Hym1 association with Kic1 increased kinase activity *in vitro* and maximal interaction coincided with HM site phosphorylation *in vivo*, we hypothesized that the Kic1-Hym1 association was sufficient for HM site phosphorylation. If this were true, then fusing Hym1 to Kic1 would constitutively drive HM site phosphorylation. Therefore, we fused Kic1-HA C-terminal to Hym1-myc to produce a Hym1-myc-Kic1-HA fusion that was expressed on a *CEN* plasmid under an *ADH1* promoter. This fusion rescued the cell separation defects of *kic1∆*, *hym1∆*, and *kic1∆ hym1∆* mutants (data not shown) demonstrating that both proteins were functional. Surprisingly, when we expressed the fusion protein in metaphase/anaphase arrested cells where both endogenous Kic1 and Hym1 were deleted, we found that Cbk1 HM site phosphorylation was not persistent throughout the release (Figure 4D). We did observe, however, that peak HM site phosphorylation occurred earlier (approximately 10-20 minutes) compared to the HM site in wild type Kic1-Hym1. We did not assess whether earlier phosphorylation of the HM site with the fusion construct is biologically significant. Nevertheless, this result suggested that there was an additional layer of control for proper HM site phosphorylation and Kic1-Hym1 association alone was insufficient to induce phosphorylation. There are two possibilities for why we did not see constitutive HM site phosphorylation. The proper Kic1-Hym1 complex may not be forming intramolecularly in the fusion construct. In this scenario, the “fusion” may still function like separate independent proteins, resulting in normal HM site phosphorylation. The other possibility is that the complex is forming correctly, but cannot interact with its substrates until other processes happen. There are other proteins in the RAM network, including Tao3 and Sog2 [27,40], that may be required to associate with the Kic1-Hym1 complex and/or Cbk1 in order to bring the components together.

## Discussion

The Hippo pathway is an ancient signaling pathway that is conserved in metazoans and has been implicated in a wide variety of cellular functions ranging from cell morphology, proliferation, and apoptosis. We have shown here that in budding yeast the MO25-related effector protein Hym1 interacts with the MST/Hippo-like kinase Kic1 to enhance its kinase activity through a conserved mechanism. This promotes direct phosphorylation of a highly conserved hydrophobic motif site on the NDR/LATS kinase Cbk1. Notably, we have discovered that the Kic1-Hym1 interaction is cell cycle regulated, suggesting that MO25 proteins can act as dynamically associating regulatory subunits. Our results indicate that after exit from mitosis Hym1 interacts with Kic1 to activate the kinase, allowing robust phosphorylation of the Cbk1 HM site, ultimately promoting transcription of cell separation genes [15]. 

Is regulation of the Kic1-Hym1 interaction responsible for temporal control of Cbk1 activation? We suggest that the dynamics of Kic1-Hym1 complex formation contributes to timing of Cbk1 HM site phosphorylation, but that it is probably not the only regulatory input. When we fused Hym1 and Kic1 we found that Cbk1 HM site phosphorylation still peaks just after mitotic exit and does not persist through the cell cycle. Thus, if we assume that this fusion protein is a constitutively assembled Kic1-Hym1 complex, we conclude that formation of this complex is necessary but not sufficient for Cbk1 HM site phosphorylation. Our results do not rule out the possibility that intramolecular interaction of the Hym1 and Kic1 parts of the fusion construct are regulated. However, we speculate that assembly of the Kic1-Hym1 module into higher order complexes with other factors facilitates Kic1’s phosphorylation of Cbk1’s HM site.

In *S. pombe*, a large scaffold protein has been shown to interact with both the Hippo kinase as well as the NDR kinase [41,42]. This scaffold protein, named *furry* in *Drosophila melanogaster*, is conserved in other metazoans and likely works in concert with the NDR kinases to control morphogenesis and proliferation [43-45]. In budding yeast this protein, called Tao3, is essential for proper HM site phosphorylation. Its deletion results in defective cell separation to the same degree of defective cell separation seen in *kic1∆*, *hym1∆*, or *cbk1∆* cells [14,27,40,46]. Two-hybrid analysis and large scale mass spectrometric identification of protein complexes indicates that Tao3 interacts with Cbk1 and Kic1 [27,47]. In the MEN, the *furry*-related protein Nud1 has been shown to be essential for recruiting the Kic1-related kinase Cdc15 and its substrate, the Dbf2-Mob1 complex, to the spindle pole body [48,49]. Thus, Tao3 may act as a scaffold that brings together the Kic1-Hym1 complex and the Mob2-Cbk1 complex. The exact mechanism of regulatory control of Tao3 and the Kic1-Hym1 module remains to be understood.

Studies of yeast Hippo signaling systems, as well as analysis of the regulation of mammalian MST kinases, suggest that the MO25-MST/Hippo complex is a broadly conserved functional module [18,50]. Intriguingly, this complex that is a crucial component of ancient Hippo signaling pathways has apparently also been incorporated into a different system that involves similar biochemical mechanisms of interaction between MO25 and a pseudokinase. In this system, the mammalian STRAD pseudokinase interacts with MO25 and LKB1, and this complex is crucial in AMPK regulation and other diverse cellular processes [31,51]. STRAD is closely related to the GCK family kinases (which include MST/Hippo kinases) and thus it probably arose from an inactivated Hippo ortholog while maintaining interaction motifs with MO25. It has been suggested that the kinase component of the MO25-STRAD evolutionary antecedent was catalytically active and regulated LKB1 by phosphorylation [31,52-54]. This ternary complex might then have retained functional importance despite mutational loss of kinase activity by STRAD. Overall, the interaction of MO25 orthologs with multiple GCK-family kinases suggests that it functions as a distinct module within diverse signaling systems.

## Supporting Information

Figure S1 **Kic1 phosphorylates the Cbk1 HM site *invitro*.** We performed ^32^P-ATP kinase assays with bacterially purified recombinant GST-Kic1(1-359)-His_6_ as the kinase and GST-Cbk1(673-756)-His_6_ as the substrate where the phosphoacceptor site Thr743 was either WT or mutated to a non-phosphorylatable residue (T743E). We detected more phosphorylation of the Cbk1 fragment when the Thr743 phosphoacceptor site was WT. We observed significant phosphorylation of the Cbk1 T743E fragment, likely due to other phosphoacceptor sites present on the fragment.
(TIF)

Figure S2 **Analysis of putative Kic1-Hym1 interaction residues.** **A**. We performed ^32^P-ATP kinase assays with different alleles (WT, D57A, L90Q, C134F, or WDF-AAA) of bacterially purified recombinant GST-Kic1(1-359)-His_6_ as the kinase and GST-Cbk1(673-756)-His_6_ as the substrate. Where indicated, we added bacterially purified recombinant full-length Hym1-His_6_. The D57A and WDF-AAA alleles of Kic1 abolished the kinase activation upon addition of Hym1. L90Q and C134F alleles retained Hym1 activation of Kic1 as WT. **B**. We performed a yeast two hybrid analysis to confirm that the WDF motif and Asp57 on Kic1 is important for Kic1-Hym1 interaction. We cloned Kic1 alleles and Hym1 into pGAD-C1 and pGBD-C1, and we coexpressed these constructs as fusion proteins with the GAL4 activation domain and DNA-binding domain, respectively. We used plasmids expressing the indicated proteins either as prey or bait alone as negative controls and wild type Kic1 and Hym1 as a positive control. We found both the WDF mutant and the D57A allele abolished Kic1-Hym1 interaction.
(TIF)
