# Supplementary figures and images for: Cell Cycle Regulated Interaction of a Yeast Hippo Kinase and Its Activator MO25/Hym1
Source: PLoS One. 2013 Oct 21;8(10):e78334. doi: 10.1371/journal.pone.0078334 (PMC3804511; doi:10.1371/journal.pone.0078334)

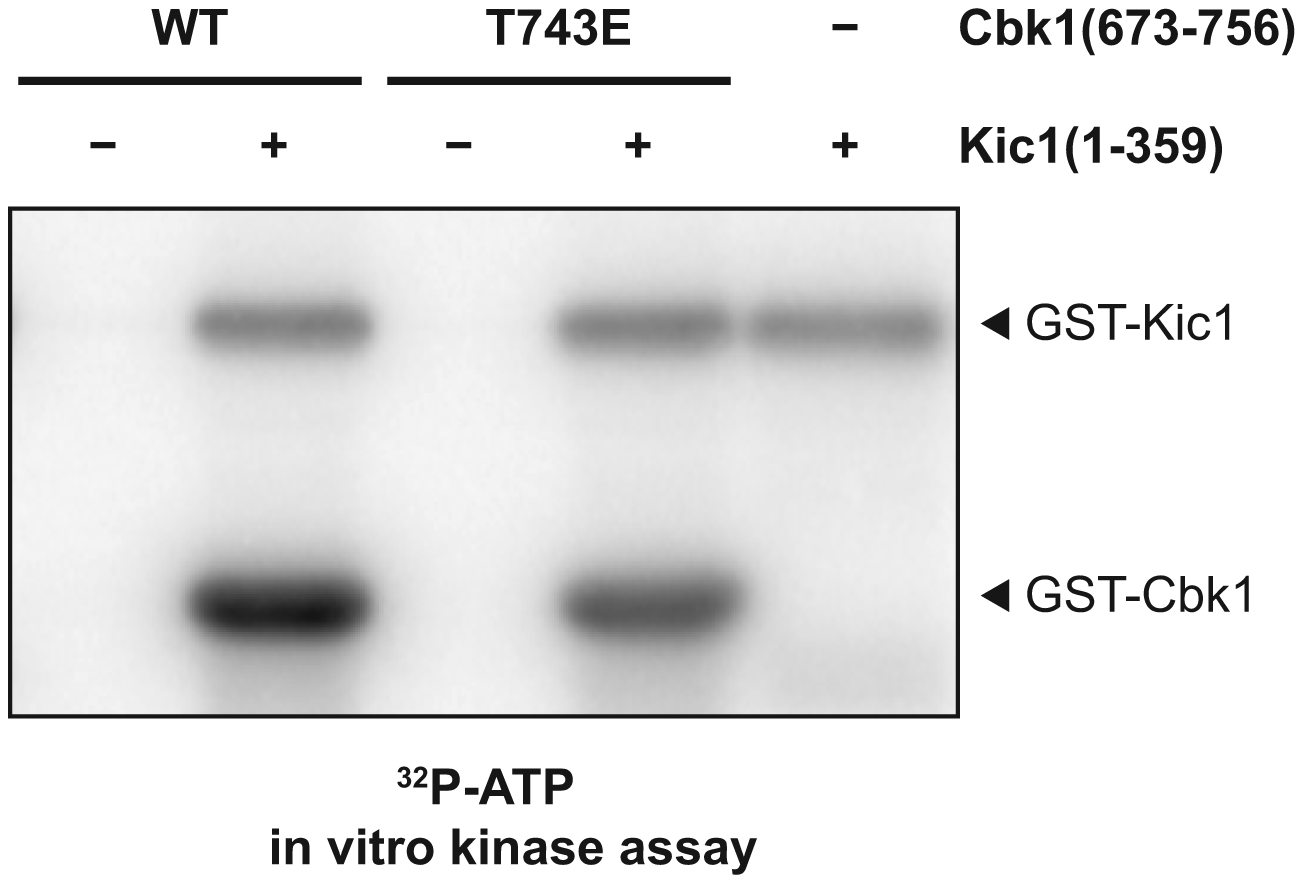

Supplement: Figure S1 — Kic1 phosphorylates the Cbk1 HM site invitro. We performed 32P-ATP kinase assays with bacterially purified recombinant GST-Kic1(1-359)-His6 as the kinase and GST-Cbk1(673-756)-His6 as the substrate where the phosphoacceptor site Thr743 was either WT or mutated to a non-phosphorylatable residue (T743E). We detected more phosphorylation of the Cbk1 fragment when the Thr743 phosphoacceptor site was WT. We observed significant phosphorylation of the Cbk1 T743E fragment, likely due to other phosphoacceptor sites present on the fragment. (TIF) [file pone.0078334.s001.tif]

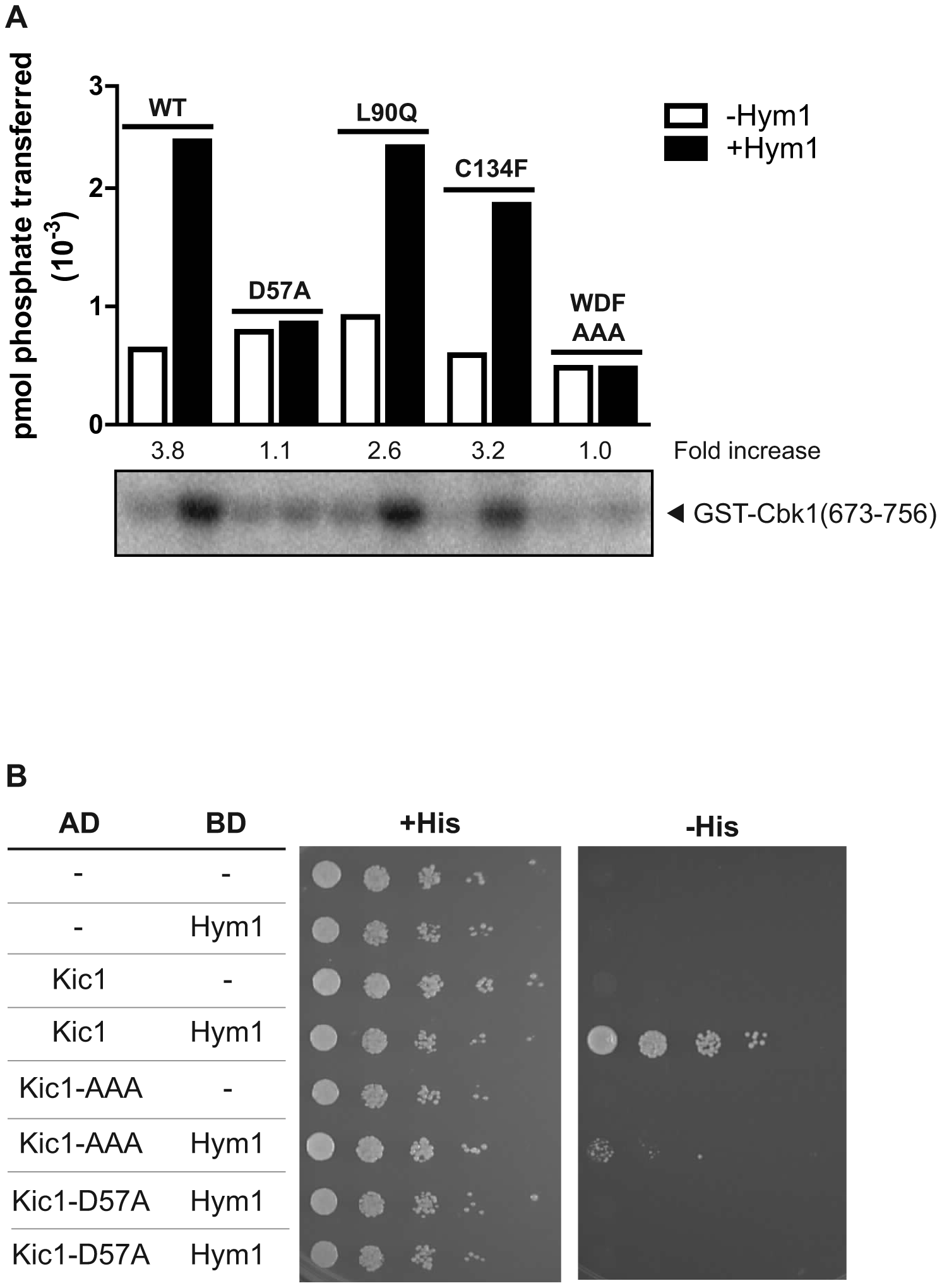

Supplement: Figure S2 — Analysis of putative Kic1-Hym1 interaction residues. A. We performed 32P-ATP kinase assays with different alleles (WT, D57A, L90Q, C134F, or WDF-AAA) of bacterially purified recombinant GST-Kic1(1-359)-His6 as the kinase and GST-Cbk1(673-756)-His6 as the substrate. Where indicated, we added bacterially purified recombinant full-length Hym1-His6. The D57A and WDF-AAA alleles of Kic1 abolished the kinase activation upon addition of Hym1. L90Q and C134F alleles retained Hym1 activation of Kic1 as WT. B. We performed a yeast two hybrid analysis to confirm that the WDF motif and Asp57 on Kic1 is important for Kic1-Hym1 interaction. We cloned Kic1 alleles and Hym1 into pGAD-C1 and pGBD-C1, and we coexpressed these constructs as fusion proteins with the GAL4 activation domain and DNA-binding domain, respectively. We used plasmids expressing the indicated proteins either as prey or bait alone as negative controls and wild type Kic1 and Hym1 as a positive control. We found both the WDF mutant and the D57A allele abolished Kic1-Hym1 interaction. (TIF) [file pone.0078334.s002.tif]
